# Self-Navigation with Compressed Sensing for 2D Translational Motion Correction in Free-Breathing Coronary MRI: A Feasibility Study

**DOI:** 10.1371/journal.pone.0105523

**Published:** 2014-08-29

**Authors:** Gabriele Bonanno, Gilles Puy, Yves Wiaux, Ruud B. van Heeswijk, Davide Piccini, Matthias Stuber

**Affiliations:** 1 Department of Radiology, University Hospital (CHUV) and University of Lausanne (UNIL), Lausanne, VD, Switzerland; 2 Center for Biomedical Imaging (CIBM), Lausanne, VD, Switzerland; 3 Institute of Electrical Engineering, École Polytechnique Fédérale de Lausanne (EPFL), Lausanne, VD, Switzerland; 4 Department of Radiology and Medical Informatics, University of Geneva (UniGE), Genève, GE, Switzerland; 5 Institute of Sensors, Signals & Systems, Heriot Watt University, Edinburgh, United Kingdom; 6 Advanced Clinical Imaging Technology, Siemens Healthcare IM BM PI, Lausanne, Switzerland; University of Washington School of Medicine, United States of America

## Abstract

**Purpose:**

Respiratory motion correction remains a challenge in coronary magnetic resonance imaging (MRI) and current techniques, such as navigator gating, suffer from sub-optimal scan efficiency and ease-of-use. To overcome these limitations, an image-based self-navigation technique is proposed that uses “sub-images” and compressed sensing (CS) to obtain translational motion correction in 2D. The method was preliminarily implemented as a 2D technique and tested for feasibility for targeted coronary imaging.

**Methods:**

During a 2D segmented radial k-space data acquisition, heavily undersampled sub-images were reconstructed from the readouts collected during each cardiac cycle. These sub-images may then be used for respiratory self-navigation. Alternatively, a CS reconstruction may be used to create these sub-images, so as to partially compensate for the heavy undersampling. Both approaches were quantitatively assessed using simulations and in vivo studies, and the resulting self-navigation strategies were then compared to conventional navigator gating.

**Results:**

Sub-images reconstructed using CS showed a lower artifact level than sub-images reconstructed without CS. As a result, the final image quality was significantly better when using CS-assisted self-navigation as opposed to the non-CS approach. Moreover, while both self-navigation techniques led to a 69% scan time reduction (as compared to navigator gating), there was no significant difference in image quality between the CS-assisted self-navigation technique and conventional navigator gating, despite the significant decrease in scan time.

**Conclusions:**

CS-assisted self-navigation using 2D translational motion correction demonstrated feasibility of producing coronary MRA data with image quality comparable to that obtained with conventional navigator gating, and does so without the use of additional acquisitions or motion modeling, while still allowing for 100% scan efficiency and an improved ease-of-use. In conclusion, compressed sensing may become a critical adjunct for 2D translational motion correction in free-breathing cardiac imaging with high spatial resolution. An expansion to modern 3D approaches is now warranted.

## Introduction

One of the major challenges in free-breathing coronary magnetic resonance imaging (MRI) is the compensation of respiratory motion. If uncorrected, respiratory motion leads to blurring or ghosting and results in decreased diagnostic value of the acquired images. To account for respiratory-induced motion, most commonly used strategies involve navigator (NAV) echoes obtained from the dome of the right hemi-diaphragm [1], [2], [3], [4]. Nevertheless, navigator techniques suffer from several limitations, including limited scan efficiency and ease-of-use problems. Respiratory self-navigation (Self-Nav) has been proposed [5], [6] as an alternative approach for respiratory motion compensation in coronary MRI. Using Self-Nav, respiratory motion parameters are extracted directly from the readouts used for imaging of the heart. These motion parameters are then used to correct for motion during image reconstruction, after scan completion. This allows for 100% scan efficiency and an improved ease-of-use, as laborious scout scanning and navigator placement are no longer required. One category of self-navigated approaches consists of methods that initially acquire a central k-space line in superior-inferior (SI) direction at the beginning of each data segment acquisition, followed by a 3D radial acquisition. From the SI projection, the position of the heart can be estimated and a one-dimensional respiratory motion correction [6], [7], [8] may be performed. With this approach, average scanning time of 3D radial coronary techniques decreased from ∼16 min (for NAV) to ∼6 min (for Self-Nav) [8]. However, similarly to the conventional NAV, the displacement of the heart in non-SI directions cannot easily be determined. Thus, to account for 2D respiratory displacement of the heart, image-based self-navigation techniques have been proposed. These 2D techniques make use of additional low-resolution Cartesian images to extract motion in multiple directions [9]. Alternatively, by using a 2D radial acquisition strategy, ghosting artifacts are no longer an issue for undersampled acquisitions and the very same projections used in the final image reconstruction may also be exploited to extract respiratory motion parameters. However, while two-dimensional motion information may be extracted from the radial k-space readouts that are acquired in each cardiac cycle, the limited amount of radial readouts per data segment provide a rather sparse representation in the Fourier domain with aliasing, or streaking, in the image domain. Therefore, simply extracting two-dimensional motion parameters from such radial data segments with the goal to augment self-navigation may not be straightforward [10], [11], [12]. Recently, compressed sensing (CS) has been widely studied to accelerate MR acquisitions [13], [14], [15], [16]. CS theory shows that it is possible to accurately reconstruct sparse MR images from only a few number of k-space measurements. Images are then reconstructed from these measurements with the use of non-linear techniques. In dynamic cine cardiac MRI, CS has recently not only been applied to accelerate image acquisition but also to support motion compensation with Cartesian [17] and non-Cartesian trajectories [17], [18], [19]. Similarly, parallel imaging [20] and a combination of CS with parallel imaging [21], [22] has been employed for respiratory motion compensation. In coronary MR angiography (MRA), CS has been used to assist final image reconstruction [23], to abbreviate scanning time for navigator gated [24] and multiple breath-hold [25] approaches, and, preliminarily, to support motion correction and final image reconstruction in a self-navigated 3D Cartesian trajectory [26]. In the present study, we hypothesized that image quality in self-navigated, volume targeted free-breathing radial coronary MRA can be improved by exploiting CS to extract accurate 2D respiratory motion parameters. Here, CS is used to reconstruct heavily undersampled images (henceforth referred to as sub-images) from the radial k-space readouts acquired during each cardiac cycle. To test this hypothesis, an image-based self-navigation technique that incorporates CS reconstruction, and allows for two-dimensional motion correction, was first implemented as a 2D imaging technique. It was then tested in healthy volunteers by acquiring images of, and then analyzing, the proximal segments of the right coronary artery. Specifically, for testing the feasibility of such an approach, targeted free-breathing in vivo coronary MRA scans were acquired with an interleaved 2D radial sampling scheme where undersampled sub-images were reconstructed either with or without CS from each data segment (interleave). In these sub-images, in-plane translational motion parameters were extracted for each cardiac cycle and motion correction was then performed in k-space for each individual sub-image. All motion corrected sub-images were then combined to create the final coronary MRA dataset. The image quality from both the CS-assisted and the non-CS assisted self-navigation techniques was quantitatively assessed and compared to that from conventional navigator gating.

## Methods

Upon written request to the authors, the data used and produced in this study can be made available under the condition that local Ethics Committee approval to share said data is obtained. Some of the algorithms utilized are already available in the public domain, which is clearly indicated below.

### Self-navigation incorporating compressed sensing

**Figure 1:**
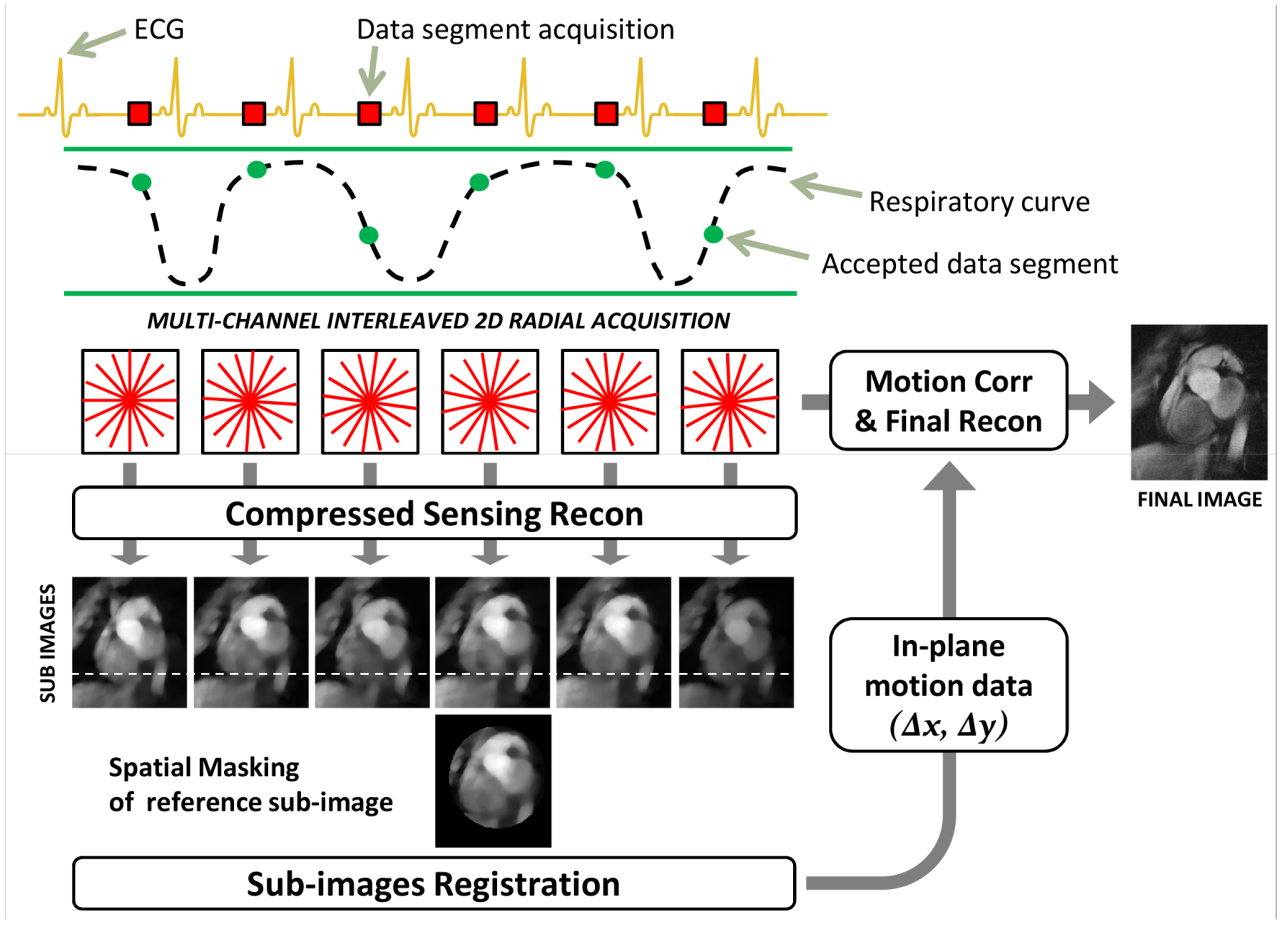
Schematic of the self-navigation method using compressed sensing (Self-Nav CS). An interleaved two-dimensional radial acquisition is performed with ECG triggering and during free-breathing. Undersampled sub-images are reconstructed from k-space data acquired during each heart-beat (interleaved red stars) using a compressed sensing algorithm based on a Total Variation prior. Motion detection is performed by registering all the sub-images to extract in-plane translation data. A binary mask is generated by the user on the reference sub-image and is used for the similarity measure computation during registration. Motion parameters are then extracted as displacements in two dimensions (Δx, Δy) and used to correct k-space data on a beat-to-beat basis prior to conventional, final image reconstruction.

In the proposed image-based self-navigation method, sub-images are generated using CS reconstruction to suppress artifacts due to undersampling. Motion is measured by co-registering all sub-images to a reference sub-image. In the present implementation, only in-plane translational displacement is corrected. A diagram that illustrates the pipeline of the technique is shown in Fig. 1. An interleaved 2D radial acquisition strategy is used for imaging, since aliasing in undersampled radial sub-images leads to incoherent noise-like artifacts that may be reduced with a non-linear iterative reconstruction [15].

#### Sub-image reconstruction

To estimate the respiratory motion parameters of the heart with self-navigation, undersampled sub-images were reconstructed from each interleave (resulting in 2.89% of Nyquist equivalent undersampling) with a) a non-linear reconstruction algorithm (Self-Nav CS method) based on a Total Variation prior [27] and with b) a conventional linear reconstruction (Self-Nav LN method) that consisted of density compensation and convolution-based gridding [28] of the radial profiles and fast Fourier transform (FFT) for comparison purposes. Because of the undersampling, the reconstruction becomes an ill-posed inverse problem and, for the Self-Nav CS method, the image is estimated through a non-linear iterative process in order to find the best match with the given data by minimizing the following functional:
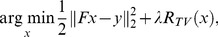
(1)


On the right hand side of Eq. 1, the first functional is a squared *L_2_*-norm of the residual between the measured noisy data ***y*** and ***Fx***. The matrix ***F*** models the acquisition and denotes the subsampled Fourier operator, implemented using the non-uniform FFT [29], [30], [31], that computes the k-space coefficients of the estimated image ***x***. The second term *R_TV_(* ***x*** *)* is a prior term used to regularize the ill-posed inverse problem. Its effect is weighted by *λ*>0, which was experimentally found to be optimal at 10^−5^. In this coronary imaging scenario, the following Total Variation prior was chosen to search for an image estimate with sparse gradient:
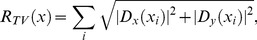
(2)where *D_x_* and *D_y_* represent the spatial derivatives of the image ***x*** at pixel ***i***, computed as the finite difference between neighboring pixels in *x* and *y* direction respectively. The underlying idea is that by minimizing the derivatives, regions with homogenous and constant intensities are recovered and streaking artifacts and noise are removed. The magnitude of the derivatives was used in order to preserve the edges on the image and to simultaneously help suppress Gibbs ringing artifacts and noise, as suggested by Block et al. [15]. The problem in Eq. 1 was solved using the method described by Beck et al. [32], [33].

#### Motion detection

Each of the sub-images obtained with the Self-Nav CS and Self-Nav LN methods, as described above, represents the heart at the position in the respiratory cycle during which the corresponding interleave was acquired. Once all sub-images were acquired, they were co-registered to extract in-plane motion parameters of each interleave. The image registration framework was developed with the open source toolkit ITK (“The Insight Segmentation and Registration Toolkit” [34]), as an iterative process where a sub-image is modified iteratively by a given transform to match a randomly chosen reference sub-image. A rigid translation transformation was used to obtain relative in-plane displacements (Δ*x*, Δ*y*) that were subsequently used for motion correction. For each iteration, the transformed sub-image was linearly interpolated onto a Cartesian grid and compared with its reference counterpart by computing the mean square error. This similarity measure represents the cost function of the iterative process, which used a gradient descent optimizer to search for a local minimum in the transform space (corresponding to the smallest difference between the two sub-images).

For an accurate image registration, a binary ROI mask with an ellipsoidal shape was applied onto the heart and used for the computation of the similarity measure only. The ROI was manually selected in order to encompass the heart only and the mask assumed values of 1 inside the ROI while 0 elsewhere. This was implemented to avoid errors in the displacement calculation step caused by both static tissues (e. g. posterior chest wall, spinal cord), and tissues that move asynchronously with the sought cardiac motion (e. g. anterior chest wall, liver).

#### Motion correction

The estimated motion parameters of each sub-image were then used to correct the radial readouts in k-space. According to the Fourier shift theorem, a radial profile in k-space can be corrected for a rigid displacement by means of a linear phase modulation Δ*β* (Eq. 3): 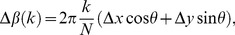
(3)where the pair (Δ*x*, Δ*y*) denotes the in-plane displacements that are projected onto the radial line with the azimuthal angle *θ. N* is the total number of data points sampled during signal-readout and *k* refers to the index of the sampling point along the readout line. Subsequently, all corrected subsets of k-space data were combined and an image was reconstructed with the conventional linear method (gridding [28] followed by FFT) for each channel individually. The final magnitude image was obtained as the square root of the sum-of-squares of all channel images.

### MR experiments – simulations

As a first validation step, in order to investigate the performance of the proposed reconstruction algorithm, volume-targeted electrocardiogram (ECG) triggered *breath-hold* images (see protocol parameters below) of the right coronary artery (RCA) were acquired in vivo in one healthy adult subject. Breath-holding was used to obtain a dataset that is free of respiratory motion [35]. Then, to quantitatively ascertain the accuracy of the sub-image reconstruction strategies used for self-navigation, the interleaved k-space data were artificially corrupted with known in-plane displacement values that were extracted from one of the free-breathing volunteer scans (see below). These in-plane displacement values were within a range of 0 to 7 mm. The corrupted image was then retrospectively corrected with the proposed self-navigation method with both linear and non-linear reconstructions. The motion detection error relative to the known respiratory displacement values (ground truth) was then evaluated for both reconstruction methods. Specifically, the Euclidean distance between the ground truth displacements and those obtained from the Self-Nav LN and Self-Nav CS methods were computed.

### MR experiments – volunteer study

In vivo experiments were performed in 12 healthy adult volunteers (3 women, 26.9±5.1 years) on a 3T clinical scanner (MAGNETOM Trio, Siemens AG, Healthcare Sector, Erlangen, Germany) with a 32-channel cardiac radiofrequency (RF) coil (Invivo, Gainesville, FL). All in vivo MR acquisitions were approved by the Institutional Review Board of the University Hospital of Lausanne (CHUV, Lausanne, Switzerland), and written informed consent was obtained from all study subjects. All clinical investigations were conducted according to the principles expressed in the Declaration of Helsinki. After survey imaging, three 2D ECG gated cine-scans with axial slice orientation were acquired at the base, mid-ventricular level and the apex of the heart. These cine images were used to visually identify the onset of the minimal coronary motion period ( = trigger delay) that was then used for the subsequent targeted coronary MRA acquisition. Using the cine frames acquired at the trigger delay time and from all 3 anatomical levels, the RCA was visually identified and the double-oblique plane of the RCA was defined as previously reported [36]. ECG-triggered images of the RCA were then acquired using either navigator gating (NAV, 4 mm gating window, no slice tracking) or free breathing without NAV. The coronary MRA imaging sequence consisted of an interleaved 2D radial gradient-echo acquisition with T_2_-preparation, fat saturation, and the following sequence parameters: 360 radial projections in k-space, 320 samples per projection, 24 interleaves, 15 projections per interleave, 320×320 matrix, 320×320 mm field-of-view (FoV), 1×1×8 mm spatial resolution, repetition time (TR)  = 8.9 ms, echo time (TE)  = 4.2 ms, TE_T2prep_ = 50 ms, RF excitation angle α = 15°, receiver bandwidth = 200 Hz/pixel. Reconstruction of all NAV and free-breathing images was performed off-line, on a stand-alone PC in MATLAB (Mathworks, Natick, MA), with C and C++ subroutines called for gridding and ITK registration. Free-breathing images were reconstructed both without any motion compensation as a post processing step (No Resp Gating) and with the reconstructions used by the Self-Nav LN and Self-Nav CS methods. During these Self-Nav data acquisitions, the navigator signal from the dome of the right hemi-diaphragm was still recorded, but was not actually used for gating or tracking.

#### Data analysis

For each subject, four different targeted images of the RCA were available for analysis (No Resp Gating, Self-Nav LN, Self-Nav CS, NAV). Since there is no ground-truth for the measurement of respiratory-induced heart motion, the four imaging strategies were compared for acquisition time, scan efficiency and image quality. Quantitative image quality assessment was performed with the Soap-Bubble software [37]. The SNR of the blood pool and the blood pool-to-myocardium contrast to noise ratio (CNR) were computed according to the formulas:
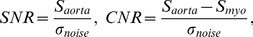
(4)where *S_aorta_* and *S_myo_* represent the mean signal value of a region of interest (ROI) manually traced inside the aorta and the myocardium of the left ventricle respectively, while *σ_noise_* is the standard deviation of the signal measured in an ROI outside the thorax. In all images, the average vessel diameter and the percent vessel sharpness (%VS) were computed for the proximal 2 cm of the RCA.

For further validation of the proposed techniques, the SI components of the in-plane displacements of the Self-Nav LN and Self-Nav CS methods were compared to the displacements measured by the diaphragmatic navigator for each subject. In particular, to obtain these self-navigated SI components, first the in-plane displacements were expressed as a function of the patient coordinate system after multiplication by the inverse of the slice rotation matrix. The projection of the thus-obtained absolute displacements onto the z-axis was then considered as the SI component.

### Statistical analysis

For the in vivo simulations, the motion detection error with respect to the known displacement values was obtained with both the Self-Nav LN and Self-Nav CS methods, for each of the 24 simulated interleaves. A paired two-tailed Student's *t*-test was used to compare two methods, with *P*<0.05 considered statistically significant.

For the volunteer study, acquisition time, scan efficiency and image quality parameters (SNR, CNR, lumen diameter and %VS) of each acquisition strategy were measured and compared. A paired two-tailed Student's *t*-test was used, with *P*≤0.008 considered statistically significant after Bonferroni correction for multiple comparisons. The correlation coefficient (CC) was then computed between the SI displacements detected by the Self-Nav methods and NAV, as a measure of synchronicity between the motion detected at the level of the heart by self-navigation and the motion of the diaphragm [38]. Hence, high CC values indicate that the motion detected by self-navigation is correlated with the respiratory cycle. Linear regression analysis was also performed to compute the regression coefficients. This measure relates to a subject-specific correction factor between respiration-induced cardiac and diaphragmatic motion. Statistical comparisons were then made using a paired two-tailed Student's *t*-test with *P*<0.05 considered statistically significant.

## Results

### Sub-image reconstruction

**Figure 2:**
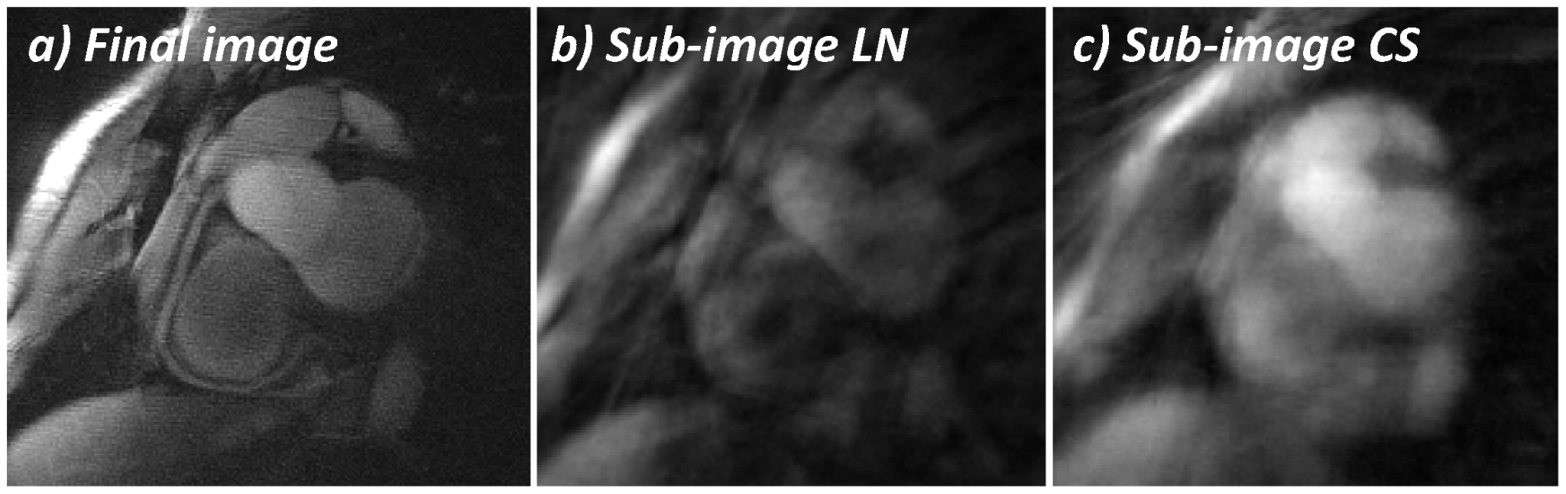
Reconstruction of in vivo sub-images. a) Representative targeted RCA from a conventional NAV acquisition. b) Sub-image obtained in the same subject from one interleave (15 radial spokes out of a total of 360) reconstructed with the linear approach (gridding followed by FFT). c) Sub-image obtained from the same interleave as in (b), however, reconstructed with the compressed sensing method based on Total-Variation.

In Fig. 2, a fully sampled image (360 acquired radial profiles) from a NAV acquisition (Fig. 2-a) is shown as a reference adjacent to two sub-image reconstructions shown in Fig. 2-b and Fig. 2-c. Both of these images were reconstructed using one interleave consisting of 15 radial profiles. The sub-image in Fig. 2-b was reconstructed with the conventional Self-Nav LN method and streaking artifacts, noise, and regional signal loss are clearly visible at the level of the heart. However, when the same k-space data were reconstructed with the Self-Nav CS method (Fig. 2-c), streaking artifacts and noise appear reduced and smoothed out at the level of the heart, while the signal appears increased and the anatomy better defined when compared to the conventional reconstruction.

### MR experiments – simulations

**Figure 3:**
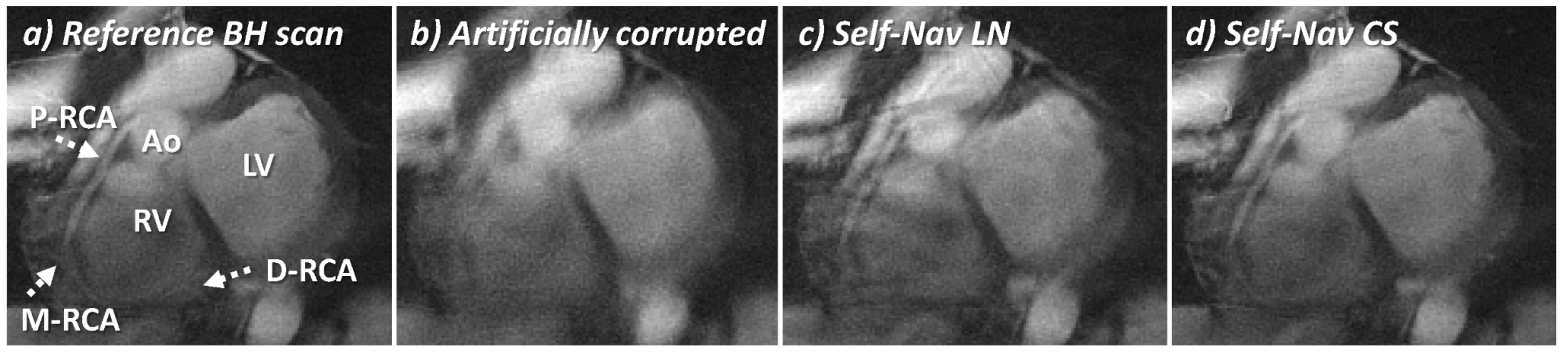
In vivo motion correction simulation. On the targeted acquisition of the right coronary artery (RCA), the following anatomical structures are visible: left ventricle (LV), right ventricle (RV), aorta (Ao), proximal (P), mid (M) and distal (D) segments of the RCA (dotted arrows). To test the performance of the self-navigation (Self-Nav) techniques, an RCA image was acquired during a breath-hold (BH) and serves as the reference standard of optimal respiratory motion suppression (a). In vivo respiratory displacement parameters were extracted at the level of a heart from a free-breathing acquisition in a healthy volunteer. These data were then used to corrupt each interleave of the standard of reference BH data simulating respiratory motion (b). These artificially corrupted data were then retrospectively corrected with the Self-Nav techniques using linear (c) and compressed sensing (CS) (d) reconstruction of the sub-images. While much of the blurring is suppressed with the Self-Nav LN method (c), residual streaking artifacts remain visible. Improved image quality and anatomy depiction were instead obtained with the Self-Nav CS method (d). This also provided more accurate motion detection, as evidenced by a 76% decrease of the error between the estimated displacements and the ground truth.

As expected, the breath-held targeted acquisition displayed in Fig 3-a does not present streaking or blurring and the RCA appears well depicted (arrows). After artificial motion corruption of the same k-space data, the reconstructed image appears blurred (Fig. 3-b) and segments of the RCA can no longer be identified. In the images reconstructed with either self-navigation technique, the coronary becomes visible again and improved quality can be appreciated. With linear reconstruction of the sub-images, much of the blurring is suppressed and the anatomy is partially restored when compared to the motion corrupted image in Fig 3-b. However, streaking artifacts originating from the surrounding structures can still be identified at the level of the mid RCA, and residual blurring at the borders of the left ventricle (LV) can be identified (Fig. 3-c). When using non-linear CS reconstruction of the sub-images, streaking artifacts are reduced and the borders of the RCA and the LV visually appear more clearly defined (Fig. 3-d). Consistent with these findings, the average error of the motion detection among all 24 sub-images was 0.38±0.29 mm when using non-linear reconstruction and 1.58±1.11 mm with linear reconstruction (*P*<0.001). Expressed as relative average error (divided by the displacement), this was 16.9±16.7% and 76.3±97.6% respectively (*P*<0.003).

### MR experiments – volunteer study

**Figure 4:**
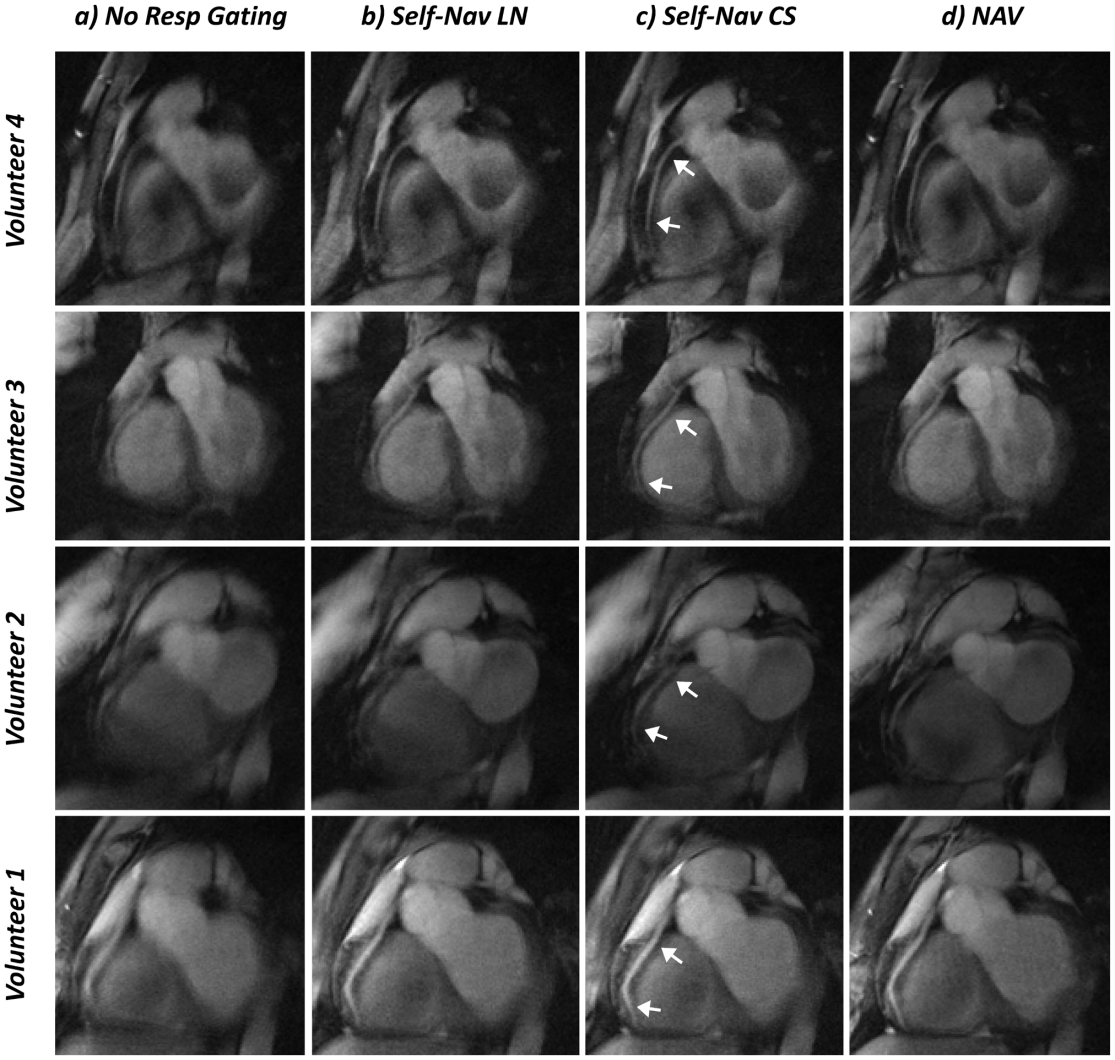
In vivo results from four healthy volunteers. a) Respiratory motion corrupted images from targeted acquisitions of the right coronary artery (RCA) without any respiratory gating. The images appear blurred and the RCAs not well depicted. When the same image data are retrospectively motion compensated using self-navigation with linear reconstruction (Self-Nav LN) of the sub-images (b), motion artifacts are suppressed and the anatomy is restored in the image with improved visibility of the RCA. c) Self-navigation with non linear reconstruction of the sub-images (Self-Nav CS) provides an improved image quality with better coronary delineation (arrows) when compared to the images obtained with the No Resp Gating and Self-Nav LN methods. The overall image quality approaches that of the standard of reference NAV acquisition in (d), albeit with a 2.8-fold reduction in scanning time.

When the acquisition was performed without respiratory gating or motion correction, the anatomy in the reconstructed images appeared blurred as shown in Fig. 4-a. The same data can be corrected for respiratory motion using the proposed self-navigation method with linear reconstruction of the sub-images. As expected, this leads to an overall sharper image where the anatomy is restored (Fig. 4-b). When using non-linear reconstruction of the sub-images, however, a further improvement in the visual delineation of the RCAs is obtained (Fig. 4-c) and the image quality approaches that of the navigator-gated images (Fig. 4-d). Consistent with these findings, the proposed Self-Nav CS method led to significantly improved vessel sharpness (36.2±10.7%) as compared to the No Resp Gating acquisition (27.9±10.4%, *P* = 0.0027) and to the Self-Nav LN approach (33.3±10.2%, *P* = 0.005). Superior vessel sharpness (38.54±10.6%) was still obtained with the NAV acquisition. However, this did not reach statistical significance when compared to Self-Nav CS (*P* = 0.22, Table 1). Simultaneously, no statistically significant difference in SNR, CNR or average vessel diameter was found among the images obtained with the three motion compensation strategies and the corrupted data. Acquisition time, scan efficiency and image quality parameters are reported in Table 1 for both gated and non-gated techniques. Relative to the duration of the NAV acquisition, the scan efficiency increased 2.8 times by using the proposed Self-Nav techniques, which led to an acquisition time reduction of 69% (*P*<0.001).

**Table 1:** In vivo results: quantitative analyses.

	*No Resp Gating*	*Self-Nav LN*	*Self-Nav CS*	*NAV*
**SNR [-]**	40.78±5.85	40.04±6.97	40.16±6.77	40.63±10.02
**CNR [-]**	26.11±4.38	25.02±6.97	25.98±5.62	27.48±5.47
**Average Diameter [mm]**	3.02±0.54	2.85±0.35	2.90±0.40	2.81±0.37
**Vessel Sharpness [%]**	27.86±10.38†	33.34±10.25†	36.20±10.66	38.54±10.59
**Acquisition Time [s]**	22.77±2.72	22.77±2.72	22.77±2.72	73.58±33.57†
**Scan efficiency [%]**	100±0	100±0	100±0	35.60±10.55†

Mean values ± one standard deviation from 12 healthy adult subjects are shown.

† indicates a significant difference when compared to the proposed Self-Nav CS technique, (Student's t-test with Bonferroni correction for multiple comparisons, P<0.008). SNR  =  signal-to-noise ratio of the blood pool; CNR  =  contrast-to-noise ratio between blood pool and myocardium.

**Figure 5:**
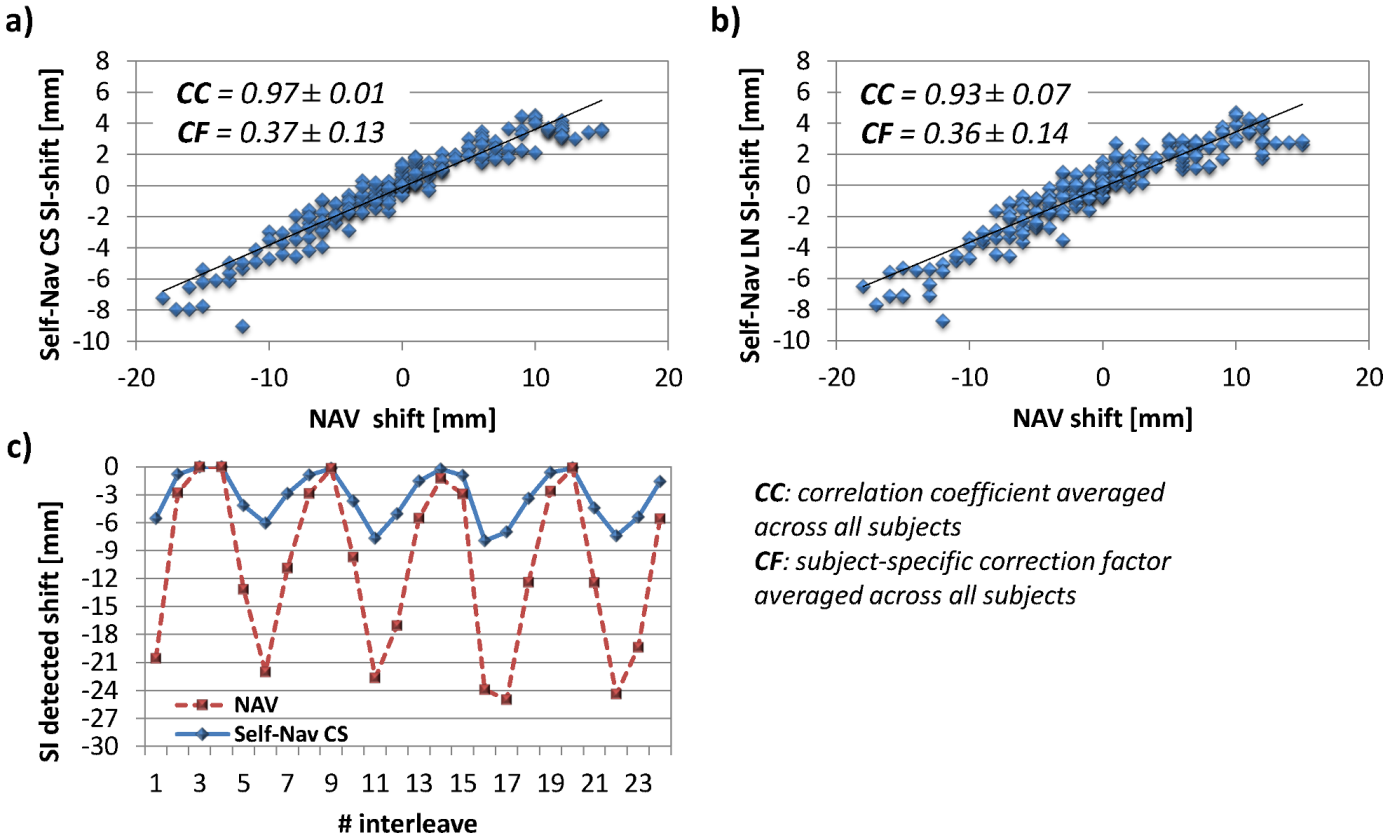
Detected motion in SI direction. Linear regression plot of the SI shifts detected in all subjects show a good correlation between NAV and Self-Nav methods: average correlation coefficient (CC) for Self-Nav CS (a) was significantly higher than that from Self-Nav LN (b) (0.97±0.01 vs 0.93±0.07, P = 0.04), indicating higher synchronicity between Self-Nav CS and NAV. Average subject-specific correction factor (CF) between diaphragmatic and respiratory-induced motion were 0.37±0.13 and 0.36±0.14 for Self-Nav CS (a) and Self-Nav LN (b), respectively. An example of the SI-component of the in-plane displacement detected with the Self-Nav CS technique (solid blue line) together with the signal detected by the diaphragmatic navigator (NAV – red dashed line) is shown in (c). In this healthy subject, the motion detected with the proposed method and the reference standard NAV show strong synchronicity, also confirmed by CC = 0.98.

When comparing the respiratory displacement regression measurements between the navigator and self-navigation, the average CC value was higher for Self-Nav CS than for Self-Nav LN (0.97±0.01 vs 0.93±0.07 respectively, *P* = 0.04). Accordingly, linear regression plots of SI shifts from all subjects show a narrower distribution of data points around the regression line when using Self-Nav CS (Fig. 5-a) and when compared to Self-Nav LN (Fig. 5-b). As a representative example, SI motion measured in a healthy adult subject with both Self-Nav CS and NAV shows high synchronicity in Fig.5-c. Averaged over all subjects, the subject-specific correction factor that relates respiration induced diaphragmatic and heart displacement was 0.36±0.14 for Self-Nav LN and 0.37±0.13 for Self-Nav CS, with the amplitude of respiratory displacement measured at the level of the heart being smaller than that measured at the level of the right hemi diaphragm.

## Discussion

A novel image-based self-navigation approach was developed for coronary MRI and preliminarily tested for feasibility with a 2D acquisition sequence. The method takes advantage of compressed sensing for improved 2D translation respiratory motion correction. While CS has primarily been exploited for reducing scanning time by undersampling and, in some applications, to simultaneously support motion compensation, in the proposed self-navigation approach, the final image is fully sampled and reconstructed with conventional linear methods and CS is solely used to reduce streaking artifacts on the aliased sub-images that are used for improved motion estimation. These sub-images are intrinsically undersampled since they are extrapolated from the individual interleaves that are a sub-set of the fully sampled acquisition. Simultaneously, in comparison to a recently described image-based navigator approach [9], no additional image acquisition is required and, as such, temporal delays no longer occur between the extraction of motion information and the actual imaging.

Several implementations that combine CS and motion estimation/correction for the purpose of enhancing reconstruction have recently been proposed for dynamic cardiac MRI [17], [18]. In coronary MRI, Akçakaya et al. [23] achieved 6-fold accelerated scanning time without significant change in image quality for targeted 3D Cartesian acquisitions. In a first attempt to support motion correction, Moghari et al. [24] proposed a combined scheme where NAV-rejected data were not reacquired to create variable density sampling for the final CS reconstruction, thus achieving gating efficiency as high of ∼80%. Recently, Prieto et al. [39] extracted undersampled sub-images from a 3D golden-step Cartesian acquisition with an iterative SENSE algorithm [40] to resolve and correct for respiratory motion. While the information from a diaphragmatic navigator was employed to populate the 3D sub-images, average acquisition times were in the order of ∼9 min.

All these studies proposed cardiac MRI techniques that exploit iterative methods to reconstruct the final images from 3D undersampled data with increased image quality, while simultaneously correcting for motion. In this contribution, we propose a modified version of a known image-based self-navigation approach [11], which incorporates non-linear reconstruction [32] to improve accuracy of motion detection and still uses linear algorithms to reconstruct a final image of the coronaries. The performance of this method as part of a 2D radial self-navigation technique was then investigated. In particular, the above-mentioned accuracy of beat-to-beat respiratory motion measurements and final image quality were measured. As a surrogate for image quality, vessel definition or vessel sharpness was quantified as previously reported [23], [39], [41], [42], [43].

As a result, this study showed that in respiratory motion simulation, based on in vivo data, CS was been shown to reduce the motion detection error of self-navigation by 76%, when compared to a purely linear reconstruction of the sub-images. This may be due to the fact that the abrupt intensity variations of the streaking artifacts that are generated due to undersampling may impair the search for the best image match during sub-image registration. In line with what was hypothesized, the use of CS reduces these artifacts and may therefore provide for a better sub-image “match”, resulting in more accurate motion detection. With the non-linear reconstruction, the error range is constrained to half of the pixel size or even lower. Accordingly, a higher image quality was found for Self-Nav CS when compared to Self-Nav LN. However, in the above-described CS framework only first order derivatives of the sub-images were used to exploit sparsity. While a sufficient image quality was achieved for the estimation of motion parameters, the reconstruction of the sub-images may still appear “patchy” because of the high degree of undersampling (4% of the full dataset). To minimize this effect, it might be useful to investigate a recently proposed CS technique that uses a weighted average of different wavelet transforms as regularization prior in order to achieve higher levels of sparsity and improved reconstruction as a result [44]. In addition, a method that takes coil sensitivities into account, as presented before [15], may also lead to a further improvements for non-linear reconstruction of the sub-images. In vivo MR experiments in 12 healthy adult volunteers suggest that the use of CS for motion detection significantly improves vessel sharpness relative to motion corrupted images and, albeit to a lesser extent, also relative to Self-Nav LN. When comparing Self-Nav CS to the reference standard NAV, acquisition time was reduced by 69% and scan efficiency increased 2.8 times, while vessel sharpness was not significantly different than in images obtained with NAV. One might expect that an image-based Self-Nav correction would be superior to any conventional NAV correction, since the respiratory-induced motion is detected in multiple dimensions and directly at the level of the heart. However, in our study, NAV vessel sharpness was still found to be superior - a result consistent with the findings of Henningsson et al. [9]. This can be attributed to the fact that both linear and non-linear Self-Nav correct for the whole range of respiratory induced cardiac displacement, while this range was significantly constrained using NAV with a gating window of 4 mm only. As a result, Self-Nav acquisitions may also be subject to trough-plane motion, whereas the narrow range of NAV acceptance may automatically prevent such displacements from adversely affecting image quality. Some combination of the two - such as using NAV to discard data from extreme respiratory positions and reconstructing the rest with Self-Nav - may prove superior. This combined approach may also be a solution for compensation of irregular respiration, which was not tested in this study. In fact, irregular breathing is expected to be characterized by a non-periodic pattern and extreme respiratory positions: while the former is taken into account by the beat-to-beat motion detection, the latter may lead to adverse effects related to amplified through-plane motion and non-rigid deformation.

Despite the substantial gain in acquisition time using self-navigation, SNR and CNR did not show significant differences among the investigated acquisition- and reconstruction strategies. In the NAV acquisitions the increase in acquisition time is not linked with an increase in SNR since many of the acquired data segments do not contribute to the final image. However, with the proposed approach this inefficiency can be avoided, while scanning time is significantly reduced. Simultaneously, image quality was shown to be comparable to that obtained with the conventional NAV acquisitions, which may be attributable to the documented and improved accuracy of the CS-assisted motion detection.

The spatial masking of the reference sub-image employed in the image registration process was used to facilitate the motion estimation task. This method ensures that the alignment is obtained at the level of the heart, minimizing adverse effects originating from signal of surrounding anatomical structures. In the current implementation, the masking is performed manually during offline reconstruction. While this allows a precise placement and adjustment of the ellipsoidal ROI around the heart only, it remains operator–dependent and may adversely affect the robustness of the method. Moreover, manual ROI placement represents a limitation for inline processing in a clinical workflow. Nevertheless, a template- and atlas based segmentation of the heart might be used as an automatic manner to define an ROI of the heart [45].

However, regardless of the accuracy of motion detection, static structures can adversely affect the quality of the final corrected image. Since the motion-correction is performed in k-space, this results in translation of the entire image. Therefore, static structures are also shifted by the same amount leading to blurring and streaking artifacts. Selective radiofrequency excitations [46] might reduce this effect and, simultaneously, improve the motion detection by only exciting the targeted moving structures. Alternatively, a region-specific image registration of the sub-images might be used to correct locally for motion, such that static structures that surround the heart undergo minimal correction and, consequently, will produce much less streaking artifacts on the heart. Methods using local-affine [47] or non-rigid [48] transformation have been adopted for this purpose and remain to be investigated. The high correlation between the SI-component of the respiratory-induced motion of the heart as detected by Self-Nav CS and the diaphragmatic navigator suggests high synchronicity between these two measurements. Simultaneously, we found an average correction factor between respiratory-induced cardiac and diaphragmatic motion of 0.37 for Self-Nav CS and 0.36 for Self-Nav LN. While this corroborates the findings by Nagel et al. [49], where a correction factor of 30% yielded improved image quality for the proximal coronary arteries, it also suggests that the respiratory displacement of the heart is attenuated when compared to that of the diaphragm. This is also consistent with other reports [38], [42]. Just as a 2D extension of self-navigation provides a theoretical improvement over a traditional one-dimensional navigation, a 3D correction may ultimately prove best, as it should better account for the three-dimensional nature of the respiratory-induced motion of the heart. This remains to be investigated, though. Wang et al. [38] have demonstrated that the SI displacement is much more significant than that in the anterior-posterior or left-right direction. Still, the slice orientation for targeted imaging of the RCA is double-oblique with the imaging plane being oriented predominantly in the foot-head direction. This slice orientation, in combination with an 8 mm slice thickness, encompasses most of the respiratory-induced motion of the heart in-plane, but through-plane motion remains unaccounted for in our current implementation. Together with the relatively large range of correction, this may contribute to an image quality that still does not surpass that of the conventional NAV technique, given that the latter accepts only a small range of respiratory motion, minimizing the effects of through-plane displacement.

One of the major limitations of our technique includes the need of the imaging plane to be in parallel with the principal direction of respiratory motion. Therefore, the utility of the proposed approach for RCA imaging was investigated as a first step. However, off-sagittal or off-coronal image plane orientations still fulfill this requirement and may enable the motion compensated acquisition of LAD and LCX as well, but this remains to be studied. Nevertheless, out of plane motion may lead to partial coverage of a coronary artery, which, in turn, may appear as a regional narrowing and a false positive result. While false positives may be preferred over false negatives, this still is a limitation, which mandates appropriate planning of both slice orientation and thickness, and which makes the approach more operator dependent. This limitation can be avoided by increasing the volumetric coverage. Therefore, an extension of this CS technique with a 3D radial imaging sequence is currently planned. Alternatively, multiple 2D image navigators may be exploited as recently reported [41] but the time delay between navigator and imaging still needs to be considered [50] as the respiratory displacement parameters are not directly extracted from the imaging data themselves. Nevertheless, while this study represents a proof of concept of this methodology, it allowed testing the motion detection strategy with breath-hold acquisitions that were used to simulate motion on in vivo data. This demonstrated improved accuracy for the proposed motion correction strategy in our 2D setting. In contrast, with a 3D acquisition scheme, a comparison with a dataset acquired during a breath-hold would not have been possible because of the required breath-hold duration. Hence, future efforts will be directed towards an extension of Self-Nav CS with a 3D acquisition, for which a CS-assisted motion detection algorithm will likely be critical to minimize motion artifacts in 3D and to maximize the diagnostic yield. A technique that makes use of CS-reconstructed sub-images to extract more accurate 3D motion parameters is currently being investigated. With the extension of this methodology to 3D, several challenges will be encountered. Specifically, with a volumetric FoV, several data segments have to be combined to reconstruct a sub-image with an adequate amount of signal and information about motion. To this end, a respiratory signal that can be obtained from diaphragmatic navigators [12] or 1D self-navigators [51] will be needed to bin different data segments into a common respiratory phase. It would also be beneficial if the binned raw data of an individual respiratory phase were uniformly distributed in k-space in order to limit streaking artifacts on the relative undersampled reconstructed sub-image. This could be obtained by using an hybrid 3D radial stack-of-star acquisition [52] or 3D radial trajectories [8], [53] that are interleaved with golden-angle steps. Simultaneously, extension with affine or non-rigid deformation [47] may also need to be integrated, as a further improvement. In conclusion, a novel image-based self-navigation approach that incorporates compressed sensing reconstruction for 2D translation motion correction in free-breathing coronary MRI has been demonstrated to provide images with an image quality similar to that obtained with conventional diaphragmatic navigators, albeit with the advantage of a 69% reduction in scanning time. Based on the above observations, the extension of the CS methodology with a 3D imaging strategy is now justified.
